# Supplementary material for: Patterns of Intron Gain and Loss in Fungi
Source: PLoS Biol. 2004 Nov 30;2(12):e422. doi: 10.1371/journal.pbio.0020422 (PMC532390; doi:10.1371/journal.pbio.0020422)
Supplement: Table S1 — Also available at http://genes.mit.edu/NielsenEtAl/. (4.3 MB ZIP). [file pbio.0020422.st001.zip › NielsenEtAl/html/305.html]

AN8681.1.NCU00568.1.MG02998.1.FG08778.1


```
 CLUSTAL W (1.82) Multiple Sequence Alignments - Introns Inserted


Sequence 1: NCU00568.1	564 aa
Sequence 2: MG02998.1	614 aa
Sequence 3: FG08778.1	566 aa
Sequence 4: AN8681.1	630 aa
Alignment Length: 705 aa
Number Identitical Residues: 110 aa
Alignment Score (without introns) 6886


MG02998.1 	---MDPQAPVVANSTKPQRFPLLETVTE--------NKGLLLLQTRMVLTAIAIIYVGAH
NCU00568.1	--MASPEASAVNATGTILPSPVPDATPYTFHWEMLLRPEVLWFDAQVIFGALAVIWLGAH
FG08778.1 	MAENATDTATLEGSFDALNSTIGSGESWLSAFSYLQDQSFLMLEIRLVLSAIGIIYLGAH
AN8681.1  	MAEVSPLAELLGQALYQFTLVKP----------------LLPTYGHVILSALFPLYIGAH
          	 :   . :  :  :                         .*    :::: *:  :::***

MG02998.1 	ATLRRPPSAAPRTSNKF--TKNKDGKKKKKKKEEAQFIEGFQASDAILFPILAGFTLVGL
NCU00568.1	GSLRRPPSAAPPES------EDKD-EKKKKSKEDEQFTEGLTASDAIMFPILAGAVLIGL
FG08778.1 	AALRRPPSAAPAKK-----------KNLGEEEDEERFSQGLEPSDAIIFPLMAGVVLVGL
AN8681.1  	ASLSRPSSAAKPPKHTDNDAIESEYDEDEEHWNNDQKMEGLAPSDALVFPLTAGATLGGL
          	.:* **.***   . . ..: ... .:  :  :: :  :*: .***::**: ** .* **

MG02998.1 	YYLIQWMDDPEFISKIISGYFSIASLFSLGKLLGDVMHFTTSFVFPNVWKDGKGNLWRFD
NCU00568.1	YYLLEWLKDPNLLNKILRGYMSFAGIAGLGKLSGDALEILTSLIFPSVWSDRSNRLYYVD
FG08778.1 	YYLIQWLKDPDILNKILRWYMSTMSIASLVSIYSHGIEVVTSLVFPRYWRGRDGRLRAVD
AN8681.1  	YLVIKYAG-ADLLNKILGFYFSQMGIFFALTFVKDALSVLRSFIFPRKYS-RGGRTWKPS
          	* ::::   .:::.**:  *:*  .:    .:  . : .  *::**  :    ..    .

MG02998.1 	SVLQRQLVRIEETTEERPAAPADGVASTDLISVEQRGFYRDESGSWRPDEKHLMPFAGPL
NCU00568.1	-------------PKRRSQVLVDPTTNQAMITDKKS------------------PFPGVF
FG08778.1 	------------QTNRSVHVCDDAGNPTTNVSTTDN------------------PFPGPF
AN8681.1  	----------------RSEPVFSVVTTSEPADIRQS------------------PLPGIF
          	                      .           .                   *:.* :

MG02998.1 	STLSCTNSTRNKLWTVRLLVKEHWTVKLGLHGFGKTEFQLTIFDVLGLVAAAAFAAAYQY
NCU00568.1	SMVRLPPGINRLAWEIRHLFTEKWTVRLAAHGVGSFKSQIKFNHLLGFLVSIAITTAYHL
FG08778.1 	SCLAFSDKIRQSGWELRGLLTRHWAVKLFIHGMGKEQGRIKFAHVVSLVMALATALIYSY
AN8681.1  	GSIPLPKLAVRALWALREALYWRAKLRVHIHRVIHLECSLSALDILSGVLALPAVAYFTF
          	. :  .    .  * :*  .  :  :::  * .   :  :.  .::. : : . .  :  

MG02998.1 	TGAT-VLSNIMGLGMCYGAFQLMSPTSFTIGTMVLAGLFVYDIVMVFYT2PYMITVATKV
NCU00568.1	VQWH-VLSNILGAAMCYAAFGMLSPTSFGIGTAVLWGLFFYDIVMVFYT2PFMITVAKKV
FG08778.1 	TTSP-LLANTLGYAMCYGSLLLISPTDFLTSTLILVGLFFYDIIMVFYT2PYMVTVATKL
AN8681.1  	VSKPWWLTNFLGFSFCYGTLQLMSPSTFVTGSLILGSLFFYDIYFVYFT~PLMVTVAKKL
          	.     *:* :* .:**.:: ::**: *  .: :* .**.*** :*::* * *:***.*:

MG02998.1 	DAPIKLTFGDPKR---------GSMLGLGDIVLPGIFMCLCLRFDLWRHYQGRITNTKQN
NCU00568.1	DAPIKLVFKSSSG---------FSMLGLGDIVVPGLVMALALRFDLYMFYKRQIQYQPVD
FG08778.1 	DVPIKLTFQAAER---------KSILGLGDIVIPGMVMALALRFDLWLHYESKIKYESTD
AN8681.1  	DVPIKLLFPRPPAPSEAPGTVSLAMLGLGDIIIPGMMVGLALRFDLYLYYKTKGMIKARS
          	*.**** *  .  .:.:..: : ::******::**:.: *.*****: .*: :      .

MG02998.1 	LASELANTENKTMTGDGAKIVVDEIGTIQTKAEYIDPQGRWGDMLWTTGLKKAKLTVGL-
NCU00568.1	ASSKQASSTDQAAS------STNEMQFRRTKAPFVESEGQWGNRFWTTKLGNLMPNPACG
FG08778.1 	LKLIEKDPSSGAFV------TRSETKHKEVKAKYVNVKGNWGDNLWTRGAFFLFGSKQVP
AN8681.1  	ENKGLG----------------------FVKPLYQPATGGWGERFWAPSARPNEP-ELVP
          	                             .*. :    * **: :*:             

MG02998.1 	---ASAQFAKTYFMAAVWGYAFGMALTISMLLTFNHGQPALLYLVPCVTGAAWLTGFVRG
NCU00568.1	AVGAATAFPKPYFYASVAGYALGMVFTLTMLQVFNHGQPALLYLVPCVTGSVWLTGLIRR
FG08778.1 	QDLAATRFRKTYFYASVAGYLLGMCVTLAMLLVFKRGQPALLYLVPGVLGSLWLTGLVRG
AN8681.1  	PYRDARSFPKTYFTASIVGYTIGMVTTLAVMQIFDHPQPALLYLVPGVLISLWGTALAKC
          	    :  * *.** *:: ** :**  *::::  *.: ********* *  : * *.: : 

MG02998.1 	EVADMWRYTED----------GSLDVKDVVVTVDSDGKVIEEAQAKG---EAADKSASGN
NCU00568.1	ELKDVWGYTED----------GSLDTKDVVVTLDADG-------------DEVRKPARDK
FG08778.1 	EIKQMWKYTED----------GSLDIIDVVVDLDGDG-------------NAVKRIGKLE
AN8681.1  	QVHEMWDFSDAEGDEDQNRVDGENDEKDRTPSSERSRGLFARIFSRSDEDEGSHKAGKVS
          	:: ::* ::: .......  .*. *  * .   : .        : ....:   : .  .

MG02998.1 	A--SDSAAEEEAAKVDHESKDKHSSQDEAKA----RSHEGHDVFMFILRAPGAEEMDDID
NCU00568.1	D--NKEAKDGDKGQKEAEKEEEETSQE-------------YDVVLFSIKAP-RPKPPKTD
FG08778.1 	DGVVDLTKDDKKEKEEDKTKEEKKGDGKEK----------HRVFMLSVDAEEETNSE---
AN8681.1  	DGKNQRLSSLENTGHKSEVKNLENSNDKTQLGGENECSKHLDLFSISIYMPRKAGLEKTR
          	 .  .   . .    . : :: ...: . . .... . .   :. : :         .  

MG02998.1 	V---------------------------------------------
NCU00568.1	----------------------------------------------
FG08778.1 	----------------------------------------------
AN8681.1  	PVGQGEVSDSAHGKENWSYVPDSKEDNEPPTKRRRRSPRHATATSE
          	  .... :.:: .... :  ..:.......:.    :.  :::::.
```
